# Use of Acupuncture for Adult Health Conditions, 2013 to 2021: A Systematic Review

**DOI:** 10.1001/jamanetworkopen.2022.43665

**Published:** 2022-11-23

**Authors:** Jennifer Allen, Selene S. Mak, Meron Begashaw, Jody Larkin, Isomi Miake-Lye, Jessica Beroes-Severin, Juli Olson, Paul G. Shekelle

**Affiliations:** 1 Veterans Health Administration, Greater Los Angeles Healthcare System, Los Angeles, California; 2 RAND Corporation, Santa Monica, California; 3 UCLA School of Public Health, Los Angeles, California; 4 Veterans Health Administration, Central Iowa Heathcare System, Des Moines

## Abstract

**Importance:**

Acupuncture is a popular treatment that has been advocated for dozens of adult health conditions and has a vast evidence base.

**Objective:**

To map the systematic reviews, conclusions, and certainty or quality of evidence for outcomes of acupuncture as a treatment for adult health conditions.

**Evidence Review:**

Computerized search of PubMed and 4 other databases from 2013 to 2021. Systematic reviews of acupuncture (whole body, auricular, or electroacupuncture) for adult health conditions that formally rated the certainty, quality, or strength of evidence for conclusions. Studies of acupressure, fire acupuncture, laser acupuncture, or traditional Chinese medicine without mention of acupuncture were excluded. Health condition, number of included studies, type of acupuncture, type of comparison group, conclusions, and certainty or quality of evidence. Reviews with at least 1 conclusion rated as high-certainty evidence, reviews with at least 1 conclusion rated as moderate-certainty evidence, and reviews with all conclusions rated as low- or very low-certainty evidence; full list of all conclusions and certainty of evidence.

**Findings:**

A total of 434 systematic reviews of acupuncture for adult health conditions were found; of these, 127 reviews used a formal method to rate certainty or quality of evidence of their conclusions, and 82 reviews were mapped, covering 56 health conditions. Across these, there were 4 conclusions that were rated as high-certainty evidence, and 31 conclusions that were rated as moderate-certainty evidence. All remaining conclusions (>60) were rated as low- or very low-certainty evidence. Approximately 10% of conclusions rated as high or moderate-certainty were that acupuncture was no better than the comparator treatment, and approximately 75% of high- or moderate-certainty evidence conclusions were about acupuncture compared with a sham or no treatment.

**Conclusions and Relevance:**

Despite a vast number of randomized trials, systematic reviews of acupuncture for adult health conditions have rated only a minority of conclusions as high- or moderate-certainty evidence, and most of these were about comparisons with sham treatment or had conclusions of no benefit of acupuncture. Conclusions with moderate or high-certainty evidence that acupuncture is superior to other active therapies were rare.

## Key Points

**Question:**

What is the certainty or quality of evidence in recent systematic reviews for use of acupuncture in adult health conditions?

**Findings:**

This systematic review identified 434 systematic reviews published since 2013; of these, 127 assessed the certainty or quality of evidence of their conclusions. Overall, 82 systematic reviews regarding 56 health conditions were mapped, and most reviews concluded the certainty of evidence was low or very low.

**Meaning:**

Despite acupuncture having been the subject of hundreds of randomized clinical trials and systematic reviews for dozens of adult health conditions, there were few conclusions that had greater than low certainty of evidence.

## Teaser

This systematic review maps the conclusions and certainty or quality of evidence regarding the outcomes of acupuncture as a treatment for adult health conditions found in systematic reviews.

## Introduction

Acupuncture is a technique that is part of a larger system of care originating in China and other Asian countries dating back to the 12th century. Acupuncture has been practiced in China for more than 3000 years and was spread to Europe and the United States from the 16th to the 19th century. The history of acupuncture research was initiated in the 18th century and developed rapidly since then.^1^ Trained practitioners stimulate specific points on the body by inserting thin needles into the skin with the intention of restoring and balancing the qi or energy of the mind and body and promoting health.^2,3^ Acupuncture has been the subject of a vast number of randomized trials and of systematic reviews of randomized trials. In an effort to categorize this evidence base for use in decision-making by policy makers and practitioners, the Department of Veterans Affairs (VA) produced an evidence map in 2014 that included systematic reviews published through 2012. An evidence map is a form of systemic review that assesses a broad field to identify the state of the evidence, gaps in knowledge, and future research needs and that presents the results in a user-friendly format, often a visual figure or graph.^4^ As all the reviews published in 2012 or earlier can be assumed to be out of date^5^ and as new evidence continues to accumulate, VA policy makers requested a new evidence map of reviews published since 2012 to answer the question, “What is the certainty of evidence in systematic reviews of acupuncture for adult health conditions?”

## Methods

This review is an extension of a report commissioned by the VA. We used the Preferred Reporting Items for Systematic Reviews and Meta-Analyses (PRISMA) standards^6^ and filed the a priori protocol with the VA Evidence Synthesis Program Coordinating Center.

### Literature Search

The literature searches used for these maps are based on the searches used for the original evidence map of acupuncture completed in 2012 and early 2013.^7^ Five databases were included in the search: PubMed, Allied and Complementary Medicine Database (AMED), Cochrane Database of Systematic Reviews (CDSR), Web of Science, and Database of Abstracts of Reviews of Effects (DARE; ending search in 2014 when DARE ceased production). See eAppendix 1 in Supplement 1 for search methods.

### Study Selection and Data Collection

Each title was screened independently by 2 authors for relevance; any article chosen by either reviewer was included in the abstract screen. Abstracts were then reviewed in duplicate, with any discrepancies resolved by group discussion. To be included, abstracts or titles needed to be about efficacy or effectiveness of acupuncture for an adult health condition and be a systematic review. A systematic review was defined as a review that had a documented systematic method for identifying and critically appraising evidence. At this stage, we also selected titles and abstracts of systematic reviews about treatments and conditions for which acupuncture might be included, eg, we included titles such as “Interventions for the Reduction of Prescribed Opioid Use in Chronic Noncancer Pain”^8^ or “Nonpharmacologic Treatments for Symptoms of Diabetic Peripheral Neuropathy: A Systematic Review.”^9^ Systematic reviews that covered other interventions were still eligible if results for acupuncture were reported separately. Interventions such as laser acupuncture, moxibustion alone, needling, traditional Chinese medicine (TCM), and fire acupuncture were excluded.

From this large collection of systematic reviews that included acupuncture as a treatment, we next restricted eligibility to reviews that used formal methods to assess the certainty (sometimes called strength or quality) of the evidence for conclusions. In general, this meant using Grading of Recommendations, Assessment, Development, and Evaluations (GRADE).^10^ However, other formal methods were also included, such as the approach used by the US Agency for Healthcare Research and Quality Evidence-based Practice Center program.^11^ To count, an included review had to both (1) state or cite the method used and (2) report the certainty (or strength or quality) of evidence for each conclusion. After applying this restriction, there were many health conditions for which there was only 1 systematic review meeting the eligibility criteria, and we used this review for the map. For some conditions, we identified more than 1 review meeting the eligibility criteria. For these conditions, we first assessed whether the reviews differed in some other feature used to classify reviews on our map (eg, a systematic review on a certain condition included only studies comparing acupuncture with sham, while another systematic review on the same condition only included studies comparing acupuncture with other active therapies). In such cases, we included both reviews on the map, with the appropriate designations (ie, vs sham and vs active therapy). If there were multiple reviews on the same condition, and they did not differ in some other feature, then we selected the systematic review that we judged as being most informative for readers. In general, this was the most recent review or the review with the greatest number of included studies. Systematic reviews otherwise meeting eligibility criteria that were not included on the map for this reason are listed in eAppendix 2 in Supplement 1. Data on study condition; number of articles in a review; intervention characteristics; comparators; conclusions; and certainty, quality, or strength of evidence were all extracted by one reviewer and then checked by a second reviewer.

### Risk of Bias and Certainty of Evidence

Risk of bias is not part of the method of an evidence map. Certainty of evidence (as determined by the original authors of the systematic review) was abstracted for each conclusion in each systematic review and tabulated.

### Evidence Map

The visual depiction uses a bubble plot format to display information on 4 dimensions: bubble size, bubble label, columns, and rows. This allowed us to provide the following types of information about each included systematic review, as follows:

- Bubble size indicates the number of articles in systematic review. Each systematic review’s bubble size is proportional to the number of primary research studies about the outcomes associated with acupuncture included in that systematic review.
- Bubble label indicates the condition. Each bubble is labeled with the condition discussed by that systematic review.
- Shapes and colors indicate intervention characteristics. Each condition is presented in the form of a shape (type of acupuncture) and color (comparators); a condition can appear more than once if systematic reviews included either different acupuncture interventions and/or different comparators.
- Rows indicate the strength (or certainty) of findings. Each condition is plotted on the map based on the certainty of evidence statement as reported in the systematic review. Many reviews report more than 1 conclusion. Thus, to keep reviews mutually exclusive, we have 3 categories: (1) all conclusions are rated as low or very low certainty, (2) at least 1 conclusion rated as moderate certainty, and (3) at least 1 conclusion rated as high or strong certainty. For reviews with multiple certainty of evidence statements, we selected the highest certainty of evidence statement.
- Columns indicate effect of acupuncture. Each condition is plotted in either benefit or no benefit for the effect of acupuncture based on the conclusion of the review.

## Results

### Study Screening

**Figure 1.**
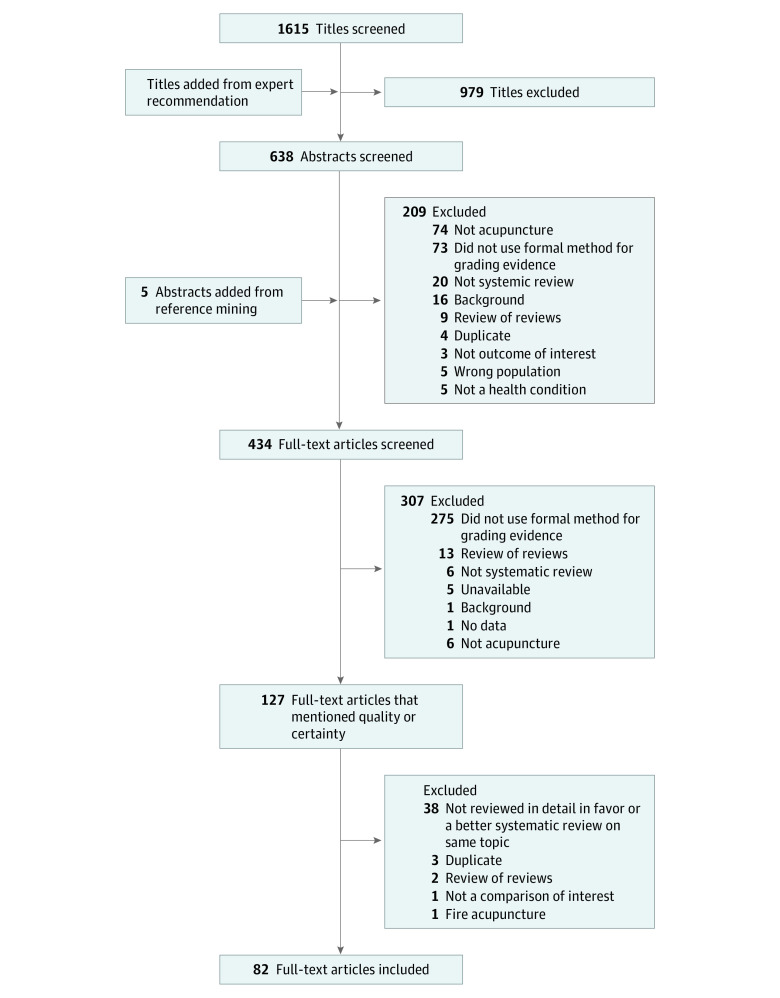
Literature Flow

The search identified 1615 citations potentially relevant to this evidence map. Including 2 publications recommended by experts, we applied the inclusion and exclusion criteria to these 1617 titles. A total of 638 abstracts were reviewed at abstract stage. From these, a total of 209 abstracts were excluded for the following reasons: not acupuncture (74), did not use formal method for grading evidence (73), background (16), not a systematic review (20), duplicate (4), not outcome of interest (3), wrong population (5), not a health condition (5), and review of reviews (9). After reference mining the cited literature in our screened full-text articles, we identified an additional 5 titles to be reviewed at the full-text stage, resulting in a total of 434 publications. From these, 307 publications were excluded for the following reasons: did not use formal method for grading evidence (275), review of reviews (13), not a systematic review (6), unavailable (5), background (1), no data (1), and not about acupuncture (6). The literature flow diagram (Figure 1) summarizes the results of the study selection process.

A total of 127 publications were retained for further review. Of these, 45 reviews were excluded from the map for the following reasons: the review overlapped a more recent or larger review which was already included on the map (38), duplicate (3), review of reviews (2), not a comparison of interest (1),^12^ and intervention was fire acupuncture (1).^13^ We included 82 publications in this map.^8,9,14,15,16,17,18,19,20,21,22,23,24,25,26,27,28,29,30,31,32,33,34,35,36,37,38,39,40,41,42,43,44,45,46,47,48,49,50,51,52,53,54,55,56,57,58,59,60,61,62,63,64,65,66,67,68,69,70,71,72,73,74,75,76,77,78,79,80,81,82,83,84,85,86,87,88,89,90,91,92,93^ A full list of publications not included on the map because they overlapped with an included review is detailed in eAppendix 2 in Supplement 1.

### Study Characteristics

The number of studies included for acupuncture in the reviews ranged from 1 to 73 studies. Thirty-seven reviews included fewer than 10 studies,^8,9,15,17,18,19,22,23,28,29,30,32,36,41,42,43,45,47,50,52,54,56,61,63,66,68,69,73,79,80,81,82,83,85,90,91,92^ 34 reviews included 10 to 25 studies,^14,20,21,25,26,27,29,30,31,33,34,35,37,38,39,40,44,48,51,53,55,58,59,64,67,70,72,74,76,78,84,86,87,93^ and 13 reviews included 25 or more studies.^16,24,46,49,57,60,62,65,71,75,77,88,89^ Nineteen of the included reviews were completed by the Cochrane Collaboration,^8,23,25,39,40,41,43,47,48,52,57,62,64,68,71,78,79,81,89^ and 3 of the included reviews were completed by the US Agency for Health Research and Quality.^28,29,50^

The country of origin for reviews varied, with the highest number of reviews originating from China (34 studies).^27,30,31,33,35,38,42,44,46,49,51,54,55,60,63,65,67,70,72,73,74,75,76,77,83,84,85,86,87,89,90,91,92,93^ Other countries included Australia (4 studies),^56,68,69,79^ Brazil (1 study),^43^ Italy (1 study),^36^ Korea (7 studies),^32,34,37,59,66,80,88^ Taiwan (1 study),^53^ the United Kingdom (1 study),^8^ and the United States (7 studies).^9,28,29,45,50,58,61^ Twenty-six publications involved teams from multiple countries,^14,15,16,17,18,19,20,21,22,23,24,25,26,39,40,41,47,48,52,57,62,64,71,78,81,82^ which included reviewers from China and the United States^14^; China and Norway^15^; China and Australia^16,17^; China (Hong Kong) and the United Kingdom^18^; Bahrain and Fiji^19^; Spain and the United Kingdom^20^; Spain, Italy, and the United Kingdom^21^; Canada and the United Kingdom^22^; Singapore and the United Kingdom^23^; Germany, the United Kingdom and the United States^24^; and Korea and the United States.^25^

Fifty-nine reviews included more than 1 type of acupuncture,^9,15,16,18,20,21,22,23,25,29,31,32,33,34,35,36,37,38,39,40,41,42,43,44,46,47,48,49,51,52,54,55,57,59,61,62,63,64,65,66,67,68,70,71,72,75,77,78,79,80,84,85,86,87,88,89,91,92,93^ while 23 reviews included only 1 type of acupuncture as the intervention.^8,14,17,19,24,26,27,28,30,45,50,53,56,58,60,69,73,74,76,81,82,83,90^ Almost all of the mapped reviews included manual or standard acupuncture as the intervention, with the exception of 4 reviews including only electroacupuncture as the intervention for the reduction of prescribed opioid use in chronic noncancer pain,^8^ for treatment of stroke,^26^ for stress urinary incontinence,^14^ and for treatment or prevention of postoperative cognitive dysfunction (POCD).^27^ A variety of comparators were included in the reviews, often involving more than 1 comparator. Forty-one reviews included more than 1 comparator and conducted separate analyses of acupuncture by comparator,^14,17,18,20,23,24,25,26,28,32,33,35,38,39,40,41,42,45,47,50,51,52,53,54,57,58,59,60,63,67,68,70,71,72,78,79,81,87,88,89,93^ while 10 reviews that had included more than 1 comparator did not conduct separate analyses.^21,29,31,43,61,65,73,75,82,92^ Twenty-one reviews included active or usual care comparators only,^16,19,27,36,37,44,46,48,49,55,56,62,74,76,77,80,84,85,86,90,91^ and 10 reviews included sham or placebo as comparators only.^8,9,15,22,30,34,64,66,69,83^

**Figure 2.**
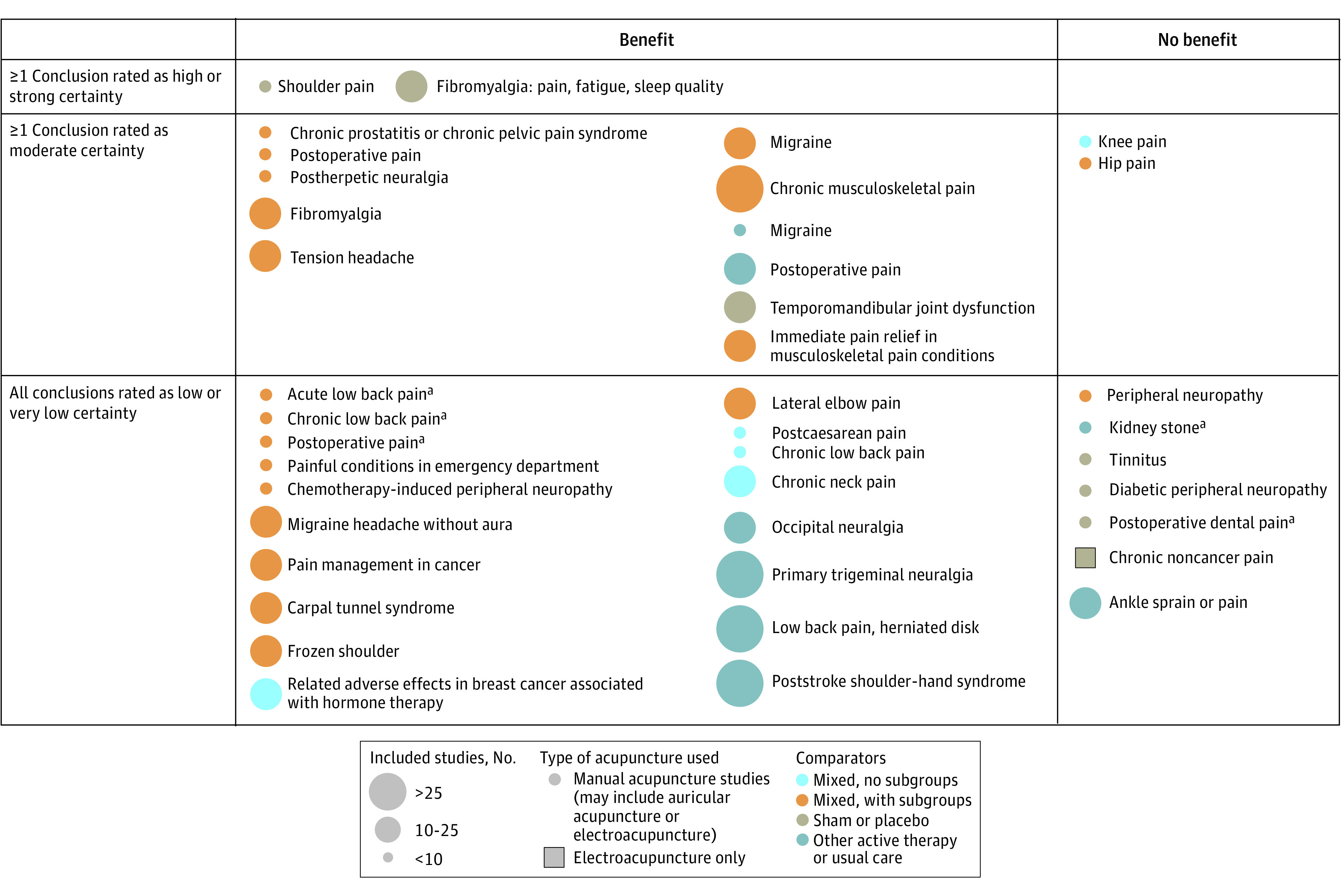
Evidence Map for Pain ^a^Review included distinct conclusions about separate conditions and comparators; therefore, it appears more than once.

**Figure 3.**
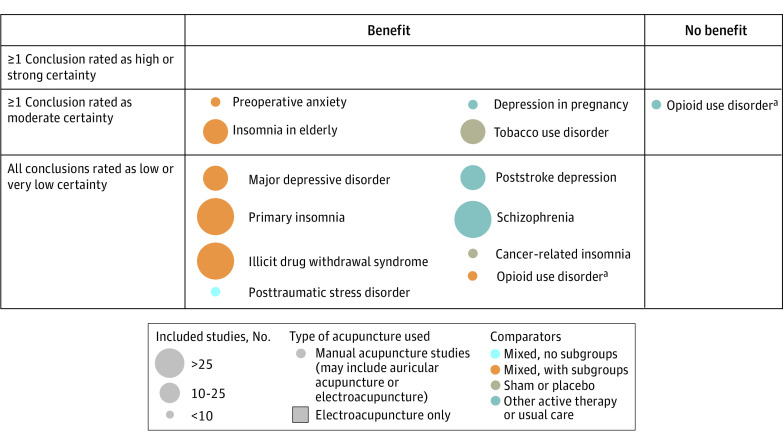
Evidence Map for Mental Health Conditions ^a^Review included distinct conclusions about separate conditions and comparators; therefore, it appears more than once.

**Figure 4.**
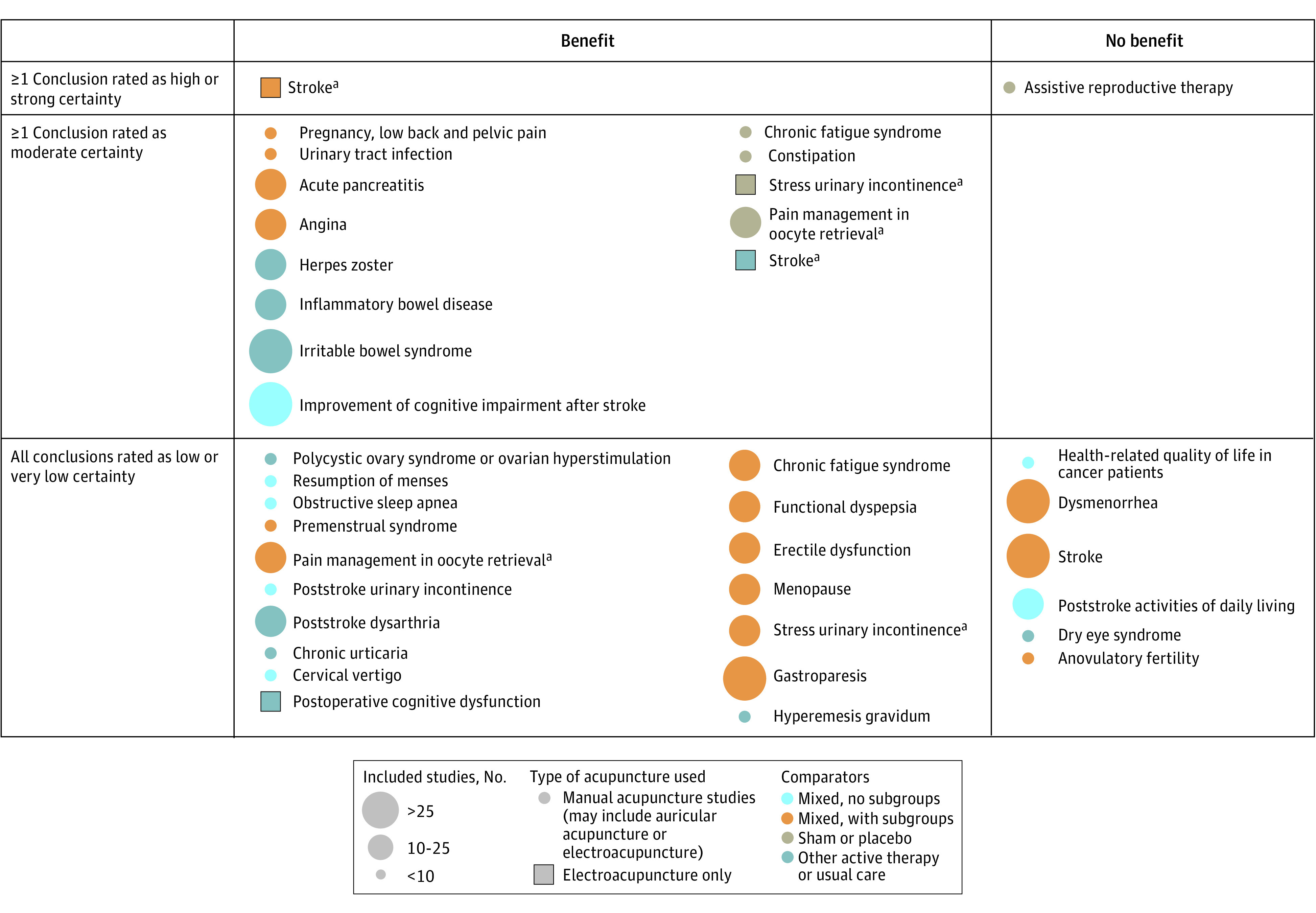
Evidence Map for Other Conditions ^a^Review included distinct conclusions about separate conditions and comparators; therefore, it appears more than once.

The included 82 reviews were categorized into 56 conditions, of which 15 conditions were further categorized into subconditions: back pain (4 studies); cancer-related pain (4 studies); chronic fatigue syndrome (2 studies); depression (4 studies); fertility (4 studies); fibromyalgia (2 studies); headache (5 studies); insomnia (3 studies); mixed pain, not specific (3 studies); osteoarthritis (2 studies); other acute pain (3 studies); postoperative pain (3 studies); shoulder pain (2 studies); stroke (3 studies); and substance use disorder (3 studies). These conditions and subconditions were then grouped by type of condition, resulting in 3 maps (Figures 2, 3, and 4). Three reviews^28,29,30^ discussed multiple conditions and thus appeared in the maps more than once.

### Evidence Maps

For ease of reading and ability to find a particular condition, the conditions were divided into 3 different maps: Pain^8,9,16,18,20,22,24,25,28,29,30,31,32,33,34,35,36,37,38,39,40,41,42,43,44,45,46,47,48,49,50,51,52,53^ (Figure 2), mental health conditions^54,55,56,57,58,59,60,61,62,63,64,65,66^ (Figure 3), and other conditions^14,15,17,19,21,23,26,27,67,68,69,70,71,72,73,74,75,76,77,78,79,80,81,82,83,84,85,86,87,88,89,90,91,92,93^ (Figure 4). Each map is structured the same way. There are 2 categories depicted in the columns: whether a conclusion of the review was that there was a benefit of acupuncture relative to a comparison treatment or that there was no benefit or no evidence of benefit of acupuncture relative to the comparison treatment. The columns are not mutually exclusive. A review could have more than 1 conclusion, and those conclusions could differ in the benefit of acupuncture.

The rows are mutually exclusive. The top row indicates that at least 1 conclusion in the review was rated by the authors of the review as having high (or strong) certainty of evidence (also sometimes called strength of evidence or quality of evidence). The middle row indicates that at least 1 conclusion was rated as moderate certainty of evidence (and none rated as high or strong, in which case it would be in the top row). The bottom column indicates that all conclusions in the review were rated as low or very low certainty of evidence.

Each conclusion was then mapped onto this framework and identified by the name of the condition or subcondition, eg, pain management in cancer, fibromyalgia, migraine. Colors were used to distinguish between the types of comparison treatments: sham or placebo, active therapies or usual care, a mix of these with no subgroup analysis presented, and a mix of these with subgroup analyses. Symbols were used to identify the few reviews specific to certain types of acupuncture, namely electro-acupuncture.

As noted previously, reviews could contain more than 1 conclusion and enter the map at different spots. For ease of presentation, we extracted and mapped all conclusions if a review had 3 or fewer conclusions. If it had more conclusions (and some reviews had >10 conclusions, eg, separate statements for each kind of acupuncture assessed, each different comparison treatment, and each different assessed outcome), we mapped the overall conclusion the authors gave to the overall body of evidence (usually found in the abstract or summary).

Most published reviews were about painful conditions: there were more mapped conclusions for painful conditions than for all other conditions combined. The number of reviews with at least 1 conclusion rated as high-certainty evidence is very small: 4. More reviews had at least 1 conclusion rated as moderate-certainty evidence (Table), but most reviews had all conclusions rated as low- or very low-certainty evidence.

**Table.** Conclusions Rated as High or Moderate Certainty of Evidence in Systematic Reviews Included in the Evidence Maps

Source	Condition (subcondition)	Certainty of evidence rating	Certainty-of-evidence conclusion
Coyle et al,^69^ 2021	Fertility (assistive reproductive therapy)	High	“Acupuncture [when given at the time of embryo transfer] did not increase the chance of live birth compared to placebo acupuncture [and] there was no statistically significant difference in the rate of miscarriage.”
Kim et al,^34^ 2019	Fibromyalgia (pain, fatigue, sleep quality)	High	“Verum acupuncture is more effective than sham acupuncture for pain relief, improving sleep quality, and improving general status in fibromyalgia syndrome posttreatment.”
Yuan et al,^30^ 2016	Shoulder pain	High	“Acupuncture was superior to sham acupuncture in terms of pain relief.”
Liu et al,^26^ 2015	Stroke	High	“[There were] better effects of electroacupuncture plus western conventional treatments for improving National Institutes of Health Stroke Scale.”
Zhang et al,^86^ 2019	Acute pancreatitis	Moderate	“Acupuncture combined with routine treatment could significantly reduce the APACHE II score, reduce the time of abdominal pain relief, and shorten the time for blood amylase to return to normal faster [compared with] routine treatment alone.”
Yang et al,^67^ 2019	Angina	Moderate	“When compared to sham treatment, acupuncture was only found to be effective in decreasing anginal attack frequency, diminishing disease-related negative emotions and increasing 6-MWT performance.”
Tong et al,^54^ 2021	Anxiety (preoperative anxiety)	Moderate	“Acupuncture therapy may be able to decrease anxiety in preoperative patients.”
Wang et al,^15^ 2014	Chronic fatigue syndrome	Moderate	“There were significant better effects in acupuncture group [compared with sham as measured by the Chalder Fatigue Score–FS score].”
Vickers et al,^24^ 2018	Chronic musculoskeletal pain	Moderate	“Acupuncture is effective for the treatment of chronic pain.”
Wang et al,^83^ 2021	Constipation	Moderate	“Acupuncture produced a significant benefit compared with polyethylene glycol and mosapride.”
Smith et al,^56^ 2019	Depression (depression in pregnancy)	Moderate	“Acupuncture compared to control [reduced] antenatal depression.”
Liu et al,^93^ 2021	Fertility (pain management in oocyte retrieval)	Moderate	“Acupuncture complex analgesic therapy is more effective than utilizing conscious sedation and analgesia or nonsteroidal anti-inflammatory drugs.”
Zhang et al,^35^ 2019	Fibromyalgia	Moderate	“Real acupuncture was significantly better than sham acupuncture in reducing pain and in improving quality of life after treatment [in the short term].”
Giovanardi et al,^36^ 2020	Headache (migraine)	Moderate	“Acupuncture is mildly more effective than medication for the prophylaxis of migraine.”
Linde et al,^40^ 2016	Headache (tension-type headache)	Moderate	“Reduction of headache frequency was much higher in groups receiving acupuncture than in control groups.”
Linde et al,^39^ 2016	Headache (migraine)	Moderate	“Acupuncture was associated with a moderate reduction of headache frequency over no acupuncture after treatment.”
Cui et al,^74^ 2021	Herpes zoster	Moderate	“Both after treatment and at follow-up, acupuncture was associated with a small but statistically significant frequency reduction over sham.”
Wang et al,^76^ 2020	Inflammatory bowel disease	Moderate	“Acupuncture may be effective in treating ulcerative colitis compared to conventional medicine (metronidazole combined with sulfasalazine).”
Kwon et al,^59^ 2020	Insomnia (insomnia in the elderly)	Moderate	“Statistically significant differences existed between acupuncture vs. acupuncture combined with relaxation and acupuncture vs. relaxation, [which suggests effectiveness for acupuncture as indicated by the Pittsburgh Sleep Quality Index total score].”
Guo et al,^77^ 2020	Irritable bowel syndrome	Moderate	“Compared with loperamide, acupuncture showed more effectiveness in weekly defecation. [Compared with dicetel,] acupuncture produced more significant effect related to the total symptom score and IBS Symptom Severity Scale.”
Xiang et al,^51^ 2017	Mixed unspecified pain (immediate pain relief in musculoskeletal pain conditions)	Moderate	“Acupuncture was associated with a greater immediate pain relief effect compared to sham acupuncture.”
Manheimer et al,^52^ 2018	Osteoarthritis (hip pain)	Moderate	“Acupuncture probably has little or no effect in reducing pain or improving function relative to sham acupuncture in people with hip osteoarthritis.”
Skelly et al,^29^ 2020	Osteoarthritis (knee pain)	Moderate	“There were no differences between acupuncture versus control interventions (sham acupuncture, waitlist, or usual care) on function in the intermediate term.”
Zhou et al,^75^ 2020	Other specific (improvement of cognitive impairment after stroke)	Moderate	“Acupuncture was effective in improving post-stroke cognitive impairment [compared with no treatment or sham].”
Franco et al,^47^ 2019	Pelvic pain (chronic prostatitis or chronic pelvic pain syndrome)	Moderate	“Acupuncture [is] likely to result in a decrease in prostatitis symptoms [compared with sham or medical treatment].”
Pei et al,^42^ 2019	Postherpetic neuralgia	Moderate	“Acupuncture may reduce pain intensity [compared with control].”
Tedesco et al,^45^ 2017	Postoperative pain	Moderate	“Acupuncture after total knee arthroplasty [was] associated with reduced and delayed opioid consumption.”
Yin et al,^44^ 2020	Postoperative pain	Moderate	“[When added to conventional medicine], acupuncture therapy may improve [the symptoms of post-operative nausea and first defecation time].”
Liddle and Pennick,^81^ 2015	Pregnancy (low back and pelvic pain)	Moderate	“[An] individual stud[y] suggests that acupuncture improved pelvic pain more than usual prenatal care.”
Zhong et al,^14^ 2020	Stress urinary incontinence	Moderate	“The effectiveness of electroacupuncture for [72-hour incontinence] was statistically significantly better than sham acupuncture.”
Liu et al,^26^ 2015	Stroke	Moderate	“[There is] a significant effect of electroacupuncture for improving [multiple outcomes] compared with western conventional treatments.”
Chen et al,^63^ 2018	Substance use disorder (opioid use disorder)	Moderate	“There was insufficient evidence to suggest better effect of acupuncture compared to medication.”
White et al,^64^ 2014	Substance use disorder (tobacco use disorder)	Moderate	“Acupuncture compared to sham acupuncture for smoking cessation [had] evidence of [a] short term effect.”
Yuan et al,^30^ 2016	Temporomandibular joint dysfunction	Moderate	“Real acupuncture [compared with sham acupuncture] has a moderate effect on musculoskeletal pain.”
Qin et al,^17^ 2020	Urinary tract infection	Moderate	“Acupuncture [compared with sham] may be prevent recurrence of urinary tract infection in women.”

Abbreviations: 6MWT, six-minute walking test; APACHE, Acute Physiology and Chronic Health Evaluation.

There are many more conclusions from systematic reviews where the original authors graded the certainty of evidence as moderate. Approximately 75% of these conclusions (23 of 31) were comparing acupuncture to sham or control acupuncture, or no treatment. Only a small number of these conclusions were about comparisons of acupuncture with usual care or other active therapies. Approximately 10% of the conclusions rated as moderate certainty (3 of 31) were findings that acupuncture was no better than the comparator. About half of these conclusions rated as moderate certainty (15 of 31) were about painful conditions or pain outcomes.

All of the remaining conclusions from the remaining reviews were judged by the original authors as being low- or very low-certainty evidence, meaning, “Our confidence in the effect estimate is limited,” “The true effect may be substantially different from the estimate of effect,” or “We have very little confidence in the effect estimate.”^10^ See eAppendix 3 in Supplement 1 for conclusions in all reviews.

### Adverse Events

In addition to maps of effectiveness outcomes, we also created a map for adverse events. Most of the 82 included reviews assessed adverse events, with 19 reviews about 20 conditions explicitly grading evidence for adverse events. eAppendix 4 in Supplement 1 presents these 20 reviews mapped by certainty-of-evidence conclusions about adverse events,^25,28,29,39,40,41,43,55,57,58,60,62,68,71,79,83,88,89,93^ of which 1 review appeared twice for 2 different conditions^29^ and 3 reviews appeared twice showing different certainty-of-evidence conclusions for different comparators.^25,29,57^

Much like our approach for maps described previously, only reviews with certainty-of-evidence conclusions specifically for adverse events were included in this map. The certainty-of-evidence conclusions were reviewed separately from conclusions for effectiveness outcomes such that it is possible to find low or very low certainty of evidence for benefit of acupuncture and high certainty of evidence for any difference in adverse outcomes in the acupuncture group.^68^

This map shows 3 categories depicted in rows: whether the certainty-of-evidence conclusion of the review for adverse events was low or very low, moderate, or high. As for columns, we listed whether there were fewer adverse events in the acupuncture group, no difference between groups, insufficient evidence to determine difference between groups, or more adverse events in the acupuncture group. A review could be mapped more than once for adverse events if different comparators had different certainty-of-evidence conclusions for adverse events. As we did for the effectiveness maps, we mapped each conclusion by name of condition or subcondition. The legend for this map is the same, with colors denoting comparators, shapes denoting types of acupuncture, and the size of bubble used to indicate the number of original research studies about acupuncture included in the review.

In the 19 mapped reviews that had included certainty-of-evidence conclusions about adverse events, most reviews reported either fewer adverse events in the acupuncture group (low or very low certainty of evidence) or no difference between groups (very low to high certainty of evidence). Only 2 reviews^25,68^ reported more adverse events in the acupuncture group. The first review about anovulatory infertility concluded with moderate certainty of evidence that “true acupuncture probably worsens adverse events compared to sham acupuncture”^68^ (ie, edema, threatened abortion, gestational diabetes, placenta previa) and concluded with very low certainty of evidence that there is insufficient data to determine differences between acupuncture and active or usual care for adverse events (ie, dizziness, nausea, subcutaneous hematoma). The second review about electro-acupuncture for carpal tunnel syndrome concluded that there were more adverse events (ie, skin bruises at the wrist or elbow due to small vessel damage) in the electro-acupuncture group (very low certainty of evidence).^25^ See eAppendix 5 in Supplement 1 for additional details about included reviews with certainty-of-evidence conclusions for adverse events.^25^

## Discussion

There is a vast literature of original randomized trials and systematic reviews of randomized trials of acupuncture as a treatment for dozens of health conditions. Despite this, the number of conditions for which authors of systematic reviews have concluded that there is at least moderate-certainty evidence regarding health outcomes associated with acupuncture was modest, and most of these involved comparisons of acupuncture with sham or control acupuncture and mostly for painful conditions. The evidence that acupuncture causes adverse health effects was rare, and reviews that compared acupuncture with usual care and included conclusions about adverse effects all concluded that acupuncture was at least as safe or safer than usual care.

Since our review was completed there has been another published study by Lu and colleagues^94^ that assessed existing systematic reviews of acupuncture across many health conditions. That effort and ours shared some characteristics, such as including only 1 review per clinical topic, in general selected because it was the largest or most recent, but it also differed in some important characteristics. Lu and colleagues^94^ considered a number of interventions that we excluded from our study, including acupressure, transcutaneous electrical nerve stimulation, and laser acupuncture; in the study by Lu and colleagues,^94^ it was a requirement that an included review have performed a quantitative meta-analysis, whereas in ours it was not; our study required a review to have performed a formal assessment of the certainty of evidence whereas in Lu and colleagues’ study^94^ this was not required; our study included data on adverse events, whereas Lu and colleagues^94^ did not; and our study included reviews with high- or moderate-certainty evidence that acupuncture did not have a beneficial effect, whereas the review by Lu and colleagues^94^ did not list any health conditions for which the conclusions were that acupuncture was not associated with improved outcomes. Nevertheless, both the study by Lu and colleagues^94^ and our study concluded that the number of clinical conditions for which there is high- or moderate-certainty of evidence is small compared with the number of conditions with low- or very low-certainty evidence.

Despite the large literature on acupuncture, most reviews concluded that their confidence in the effect was limited. Thus, the most important research need is for better evidence to move these certainty-of-evidence assessments upward, such that clinicians, patients, and policy makers can have more confidence that acupuncture does, or does not, have benefit for a certain health condition. Studies comparing acupuncture with placebo or sham are probably not the priority for reasons stated in the Limitations section; rather, the priority should be studies comparing acupuncture with other recommended, accepted, or active therapies for the condition. Facilitating new studies of adequate rigor that mitigate the limitations of existing studies sufficient to raise the certainty of evidence would be the best way to try and expand the access of acupuncture to the patients most likely to benefit from it. In the 9 years covered by this update, we identified 434 new systematic reviews of acupuncture. This compares to an approximately equal number of new randomized clinical trials of acupuncture published in the same time period and included in the systematic reviews on our map. Thus, researchers interested in acupuncture are producing about as many systematic reviews (that generally conclude the certainty of evidence is low or very low) as new randomized clinical trials needed to raise the certainty of evidence. This seems to be a mismatch between resources and need. The field of acupuncture would be best moved forward with resources devoted to producing more high quality randomized clinical trials and producing fewer new systematic reviews.

### Limitations

This study has limitations. The first, common to all systematic reviews, is that we may have not identified all the potentially eligible evidence. If a systematic review was published in a journal not indexed in any of the 5 databases we searched and we did not identify it as part of our search of references of included studies, then we would have missed it. The recent review by Lu and colleagues^94^ identified some Chinese-language articles that we did not identify because they are not indexed in any of the 5 databases we searched. The second limitation of evidence maps is that we did not independently evaluate the source evidence; in other words, we took the conclusions of the authors of the systematic review at face value. Third, one limitation to assessing the outcomes of acupuncture is the variation (and controversy) with which sham acupuncture is designed. Some studies defined sham as standard needling technique at nonactive points; some included shallow needling in both active and nonactive points; and more contemporarily, nonpenetrating needles used at both active and nonactive points. One of the major controversies of the use of sham as an inert comparator was that the unintended physiologic effects beyond placebo were not considered^95^; thus, the exact mechanism by which acupuncture is effective is unclear when compared with sham acupuncture. The uncertainty around what is considered sham acupuncture and the lack of clear understanding of the exact mechanism by which acupuncture is effective compared with sham makes conclusions about the outcomes associated with acupuncture compared with sham more challenging to interpret than, eg, the comparison of a pharmaceutical intervention with placebo, in which case the placebo is assumed with confidence to be inert.

## Conclusions

In this study, the number of conditions for which authors of systematic reviews concluded that there was at least moderate-certainty evidence regarding health outcome associations of acupuncture was modest. Most of these involved comparisons of acupuncture with sham or control acupuncture, and then mostly for painful conditions.

## Notes

Supplement 1. **eAppendix 1.** Search Strategies **eAppendix 2.** Excluded Studies Meeting Eligibility Criteria That Were Not Included on the Map **eAppendix 3.** Conclusions From Systematic Reviews Included in the Evidence Map **eAppendix 4.** Evidence Map for Adverse Events **eAppendix 5.** Certainty of Evidence Conclusions for Adverse Events in Reviews Included in Map

Supplement 2.
Data Sharing Statement
