# Errors in Figure 3 and Supplement 1

**DOI:** 10.1001/jamanetworkopen.2023.3459

**Published:** 2023-02-24

In the Original Investigation titled “Use of Acupuncture for Adult Health Conditions, 2013 to 2021: A Systematic Review,”^1^ published November 23, 2022, there were errors in Figure 3 and Supplement 1. In Figure 3, the indicator for depression in the section of all conclusions rated as low or very low certainty was missing. In Supplement 1, the number of studies included in the row for Zhou et al^2^ in eAppendix 3 should have read 37. This article has been corrected.^1^
