# Supplementary material for: Use of Acupuncture for Adult Health Conditions, 2013 to 2021: A Systematic Review
Source: JAMA Netw Open. 2022 Nov 23;5(11):e2243665. doi: 10.1001/jamanetworkopen.2022.43665 (PMC9685495; doi:10.1001/jamanetworkopen.2022.43665)
Supplement: Supplement 1. — eAppendix 1. Search Strategies eAppendix 2. Excluded Studies Meeting Eligibility Criteria That Were Not Included on the Map eAppendix 3. Conclusions From Systematic Reviews Included in the Evidence Map eAppendix 4. Evidence Map for Adverse Events eAppendix 5. Certainty of Evidence Conclusions for Adverse Events in Reviews Included in Map [file jamanetwopen-e2243665-s001.pdf]

Supplemental Online Content

Allen J, Mak SS, Begashaw M, et al. Use of acupuncture for adult health conditions, 2013 to 2021: a systematic review. *JAMA Netw Open.* 2022;5(11):e2243665. doi:10.1001/jamanetworkopen.2022.43665

eAppendix 1. Search Strategies

eAppendix 2. Excluded Studies Meeting Eligibility Criteria That Were Not Included on the Map

eAppendix 3. Conclusions From Systematic Reviews Included in the Evidence Map

eAppendix 4. Evidence Map for Adverse Events

eAppendix 5. Certainty of Evidence Conclusions for Adverse Events in Reviews Included in Map

This supplemental material has been provided by the authors to give readers additional information about their work.

© 2022 Allen J et al. *JAMA Network Open.*

eAppendix 1. Search Strategies

PubMed
1 March 2013-present; English
Search Run: 22 April 2021
Acupuncture
AND
systematic[sb]
AND
("2013/03/01"[PDat] : "3000/12/31"[PDat])

Results: 1163

CDSR
2013-present; English
Search Run: 22 April 2021
acupuncture:ti,ab,kw

Results: 9

DARE (via CRD)
Publication Year 2013 – 2021 (However – ends at 2014 when DARE ceased production)
Any Field: acupuncture
AND
Title: systematic review

Results: 17

AMED (via Dialog)
1 March 2013 – 22 April 2021; English
ti(acupuncture) AND ("systematic review")
OR
ab(acupuncture) AND ("systematic review")

Results: 18

Web of Science
2013-22 April 2021 | Review | Article | English Language
search executed 29 September 2022
TS=(acupuncture)
AND
TS=("systematic review*")

Results: 408

© 2022 Allen J et al. *JAMA Network Open.*

eAppendix 2. Excluded Studies Meeting Eligibility Criteria That Were Not Included on the Map

1. Amaral, L.K.B., et al., Efficacy of conservative therapy in older people with nonspecific low back pain: A systematic review with meta-analysis and GRADE recommendations. Arch Gerontol Geriatr, 2020. 90: p. 104177.

2. Asher, G.N., et al., Comparative Benefits and Harms of Complementary and Alternative Medicine Therapies for Initial Treatment of Major Depressive Disorder: Systematic Review and Meta-Analysis. J Altern Complement Med, 2017. 23(12): p. 907-919.

3. Bisson, J.I., et al., Non-pharmacological and non-psychological approaches to the treatment of PTSD: results of a systematic review and meta-analyses. Eur J Psychotraumatol, 2020. 11(1): p. 1795361.

4. Boelig, R. C., S. J. Barton, G. Saccone, A. J. Kelly, S. J. Edwards and V. Berghella. Interventions for treating hyperemesis gravidarum: a Cochrane systematic review and meta-analysis. J Matern Fetal Neonatal Med, 2018. 31(18): p. 2492-2505.

5. Boelig RC, Barton SJ, Saccone G, Kelly AJ, Edwards SJ, Berghella V. Interventions for treating hyperemesis gravidarum. Cochrane Database of Systematic Reviews. 2016(5).

6. Cheong, Y.C., et al., Acupuncture and assisted reproductive technology. Cochrane Database Syst Rev, 2013(7): p. Cd006920.

7. Close, C., et al., A systematic review investigating the effectiveness of Complementary and Alternative Medicine (CAM) for the management of low back and/or pelvic pain (LBPP) in pregnancy. J Adv Nurs, 2014. 70(8): p. 1702-16.

8. Dai, L., et al., Acupuncture and Derived Therapies for Pain in Palliative Cancer Management: Systematic Review and Meta-Analysis Based on Single-Arm and Controlled Trials. J Palliat Med, 2021.

9. Deare, J.C., et al., Acupuncture for treating fibromyalgia. Cochrane Database Syst Rev, 2013. 2013(5): p. Cd007070.

10. Gao, R., et al., Acupuncture and clomiphene citrate for anovulatory infertility: a systematic review and meta-analysis. Acupunct Med, 2020. 38(1): p. 25-36.

11. Gutke, A., et al., Treatments for pregnancy-related lumbopelvic pain: a systematic review of physiotherapy modalities. Acta Obstet Gynecol Scand, 2015. 94(11): p. 1156-67.

© 2022 Allen J et al. *JAMA Network Open.*

12. He, Y., et al., Clinical Evidence for Association of Acupuncture and Acupressure With Improved Cancer Pain: A Systematic Review and Meta-Analysis. JAMA Oncol, 2020. 6(2): p. 271-278.

13. Hou, S., et al., Treatment of Chemotherapy-Induced Peripheral Neuropathy: Systematic Review and Recommendations. Pain Physician, 2018. 21(6): p. 571-592.

14. Huang, J.F., et al., Can Acupuncture Improve Chronic Spinal Pain? A Systematic Review and Meta-Analysis. Global Spine J, 2021: 11(8): p.1248-1265.

15. Jo, J., Y.J. Lee, and H. Lee, Acupuncture for polycystic ovarian syndrome: A systematic review and meta-analysis. Medicine (Baltimore), 2017. 96(23): p. e7066.

16. Kizhakkeveettil, A., K. Rose, and G.E. Kadar, Integrative therapies for low back pain that include complementary and alternative medicine care: a systematic review. Glob Adv Health Med, 2014. 3(5): p. 49-64.

17. Kolber, M.R., et al., PEER systematic review of randomized controlled trials: Management of chronic low back pain in primary care. Can Fam Physician, 2021. 67(1): p. e20-e30.

18. Kwan I, Wang R, Pearce E, Bhattacharya S. Pain relief for women undergoing oocyte retrieval for assisted reproduction. *Cochrane Database Syst Rev*. May 15 2018;5(5):Cd004829.

19. Lan, L., et al., Acupuncture for functional dyspepsia. Cochrane Database Syst Rev, 2014(10): p. Cd008487.

20. Li, C., et al., The response-time relationship and covariate effects of acupuncture for chronic pain: A systematic review and model-based longitudinal meta-analysis. Eur J Pain, 2020. 24(9): p. 1653-1665.

21. Metcalf, O., et al., Efficacy of Fifteen Emerging Interventions for the Treatment of Posttraumatic Stress Disorder: A Systematic Review. J Trauma Stress, 2016. 29(1): p. 88-92.

22. Mitchell, C., et al., Interventions for dysarthria due to stroke and other adult-acquired, non-progressive brain injury. Cochrane Database Syst Rev, 2017. 1(1): p. Cd002088.

23. Mu, J., et al., Acupuncture for chronic nonspecific low back pain. Cochrane Database Syst Rev, 2020. 12: p. Cd013814.

24. Mulla, S.M., et al., Management of Central Poststroke Pain: Systematic Review of Randomized Controlled Trials. Stroke, 2015. 46(10): p. 2853-60.

25. Nascimento, P., et al., Effectiveness of interventions for non-specific low back pain in older adults. A systematic review and meta-analysis. Physiotherapy, 2019. 105(2): p. 147-162.

© 2022 Allen J et al. *JAMA Network Open.*

26. Seo, S.Y., et al., Effectiveness of Acupuncture and Electroacupuncture for Chronic Neck Pain: A Systematic Review and Meta-Analysis. Am J Chin Med, 2017. 45(8): p. 1573-1595.

27. Smith, C.A., et al., Acupuncture or acupressure for pain management during labour. Cochrane Database Syst Rev, 2020. 2(2): p. Cd009232.

28. Sridharan, K. and G. Sivaramakrishnan. Interventions for treating nausea and vomiting in pregnancy: a network meta-analysis and trial sequential analysis of randomized clinical trials. Expert Review of Clinical Pharmacology, 2018. 11(11): p.1143-1150.

29. Tang, H., et al., Acupuncture for Lateral Epicondylitis: A Systematic Review. Evid Based Complement Alternat Med, 2015. 2015: p. 861849.

30. Trinh, K., et al., Acupuncture for neck disorders. Cochrane Database Syst Rev, 2016(5): p. Cd004870.

31. Wahbeh, H., et al., Complementary and Alternative Medicine for Posttraumatic Stress Disorder Symptoms: A Systematic Review. J Evid Based Complementary Altern Med, 2014. 19(3): p. 161-175.

32. Wu, J., D. Chen, and N. Liu, Effectiveness of acupuncture in polycystic ovary syndrome: A systematic review and meta-analysis of randomized controlled trials. Medicine (Baltimore), 2020. 99(22): p. e20441.

33. Xiang, Y., et al., Evidence of efficacy of acupuncture in the management of low back pain: a systematic review and meta-analysis of randomised placebo- or sham-controlled trials. Acupunct Med, 2020. 38(1): p. 15-24.

34. Xie, Z.Y., et al., The effects of acupuncture on pregnancy outcomes of in vitro fertilization: a systematic review and meta-analysis. BMC Complement Altern Med, 2019. 19(1): p. 131.

35. Yu, C., et al., Effectiveness of acupuncture for angina pectoris: a systematic review of randomized controlled trials. BMC Complement Altern Med, 2015. 15: p. 90.

36. Yuan, Q.L., et al., Traditional Chinese medicine for neck pain and low back pain: a systematic review and meta-analysis. PLoS One, 2015. 10(2): p. e0117146.

37. Zhong, Y., et al., Acupuncture in improving endometrial receptivity: a systematic review and meta-analysis. BMC Complement Altern Med, 2019. 19(1): p. 61.

38. Zhou, Y., et al., Effectiveness of Acupuncture for Lateral Epicondylitis: A Systematic Review and Meta-Analysis of Randomized Controlled Trials. Pain Res Manag, 2020. 2020: p. 8506591

© 2022 Allen J et al. *JAMA Network Open.*

eAppendix 3. Conclusions From Systematic Reviews Included in the Evidence Map

Acute Pancreatitis

Author, year	Sub-condition	Conclusion	Certainty of Evidence	Total Number of Studies Included for Acupuncture
Zhang et al,[86] 2019	None	Acupuncture combined with RT may be effective for improving total effective rate, mortality during treatment, time to abdominal pain relief with ulinastatin, abdominal pain score, abdominal distension score, time to resuming diets, IL-6, TNF-alpha, CRP, and length of hospital stay.	Very Low	12
		Acupuncture combined with routine could significantly reduce the APACHE II score, reduce the time of abdominal pain relief, and shorten the time for blood amylase to return to normal faster compared to routine care alone.	Moderate	

Angina

Author, year	Sub-condition	Conclusion	Certainty of Evidence	Total Number of Studies Included for Acupuncture
Yang et al,[67] 2019	None	Compared with sham acupuncture, acupuncture may be associated with improving average pain intensity, 6-Min Walk Test, anxiety symptoms and depression symptoms.	Very Low to Moderate	17

Ankle Pain

Author, year	Sub-condition	Conclusion	Certainty of Evidence	Total Number of Studies Included for Acupuncture
Kim et al,[48] 2014	None	We are unable to conclude whether acupuncture is more effective than other standard methods for the treatment of ankle sprains in adults because of the very low quality of the available evidence. Because the adverse effects of acupuncture treatment were not described in most of the studies, we are also unable to draw any conclusions about the safety of acupuncture.	Very Low	19

© 2022 Allen J et al. *JAMA Network Open.*

Anxiety

Author, year	Sub-condition	Conclusion	Certainty of Evidence	Total Number of Studies Included for Acupuncture
Tong et al,[54] 2021	Pre-Operative Anxiety	Acupuncture therapy, compared with sham therapy, significantly reduced the STAI-S score for patients with preoperative anxiety.	Very low to Moderate	5

Back Pain

Author, year	Sub-condition	Conclusion	Certainty of Evidence	Total Number of Studies Included for Acupuncture
Chou et al,[50] 2017	Chronic Low Back Pain (Radicular Back Pain)	Acupuncture vs sham acupuncture: moderate magnitude of effect for pain; no effect for function<br>Acupuncture vs no acupuncture: moderate magnitude of effect for pain; moderate for function	Low	9
Chou, 2017[50]	Acute Low Back Pain	Acupuncture vs sham small magnitude of effect for pain; no effect for function	Low	9
Skelly et al,[29] 2020	Chronic Low Back Pain	Acupuncture was associated with a small improvement in short-term function compared with sham acupuncture or usual care; there was no difference between acupuncture and controls in intermediate-term or long-term function.<br>Acupuncture was associated with small improvements in short-term and long-term pain compared with sham acupuncture, usual care, an attention control, or a placebo intervention, but there was no difference in intermediate-term pain.	Low<br>Low	8
Tang et al,[49] 2018	Low Back Pain - Herniated Disc	Acupuncture was better than traction and diclofenac sodium at improvements in VAS pain.	Very Low	30

Cancer-related Pain

Author, year	Sub-condition	Conclusion	Certainty of Evidence	Total Number of Studies Included for Acupuncture
Hu et al,[33] 2016	Pain Management in Cancer	Acupuncture plus drug therapy is more effective than conventional drug therapy alone, but acupuncture alone is not more effective than conventional drug therapy.<br>Acupuncture is not more effective than sham acupuncture.	Very Low	20

© 2022 Allen J et al. *JAMA Network Open.*

Author, year	Sub-condition	Conclusion	Certainty of Evidence	Total Number of Studies Included for Acupuncture
Hwang et al,[32] 2020	Chemotherapy-induced Peripheral Neuropathy	Acupuncture was more effective than pharmacological treatment.	Low	5
Lin et al,[73] 2019	Health-related Quality of Life in Cancer Patients	Acupuncture has no effect on health-related quality of life.	Low	4
Yuanqing et al,[31] 2020	Hormone Therapy-Related Side Effects in Breast Cancer Patients	Acupuncture is a moderately appropriate alternative therapy for hormone therapy-related side effects in breast cancer patients.	Low	20

Carpal Tunnel Syndrome

Author, year	Sub-condition	Conclusion	Certainty of Evidence	Total Number of Studies Included for Acupuncture
Choi et al,[25] 2018	None	No clear difference in acupuncture vs sham- short term follow-up: 8 weeks / 3 months	Very Low to Low	10
		No clear difference in clinical improvement between acupuncture vs oral corticosteroid-short term follow-up: 4 weeks / 13 months	Very Low	
		Better clinical improvement with acupuncture vs corticosteroids in long-term follow-up: 7 months / 13 months	Very Low	
		No clear difference in rates of improvement in acupuncture vs vitamin B12 - short term follow-up	Very Low	
		No clear difference in rates of improvement in electro-acupuncture vs night splints - short term follow-up	Very Low	
		There was more clinical improvement in acupuncture vs ibuprofen-short term follow-up: 4 weeks	Very Low	

© 2022 Allen J et al. *JAMA Network Open.*

Cervical Vertigo

Author, year	Sub-condition	Conclusion	Certainty of Evidence	Total Number of Studies Included for Acupuncture
Hou et al, [92] 2017	None	Acupuncture appeared to be a promising therapeutic approach for cervical vertigo.	Very low to low	9

Chronic Fatigue Syndrome

Author, year	Sub-condition	Conclusion	Certainty of Evidence	Total Number of Studies Included for Acupuncture
Wang et al,[15] 2014	None	There were significant better effects in acupuncture group compared with sham as measured by the Chalder's Fatigue Scale (physical) score.	Low to Moderate	7
Zhang et al,[70] 2019	None	In summary, acupuncture appears more effective than sham acupuncture and Chinese herbal medicine for the treatment of CFS.	Very Low to Low	13

Chronic Musculoskeletal Pain

Author, year	Sub-condition	Conclusion	Certainty of Evidence	Total Number of Studies Included for Acupuncture
Vickers et al,[24] 2018	None	Acupuncture is effective for the treatment of chronic pain compared with sham and control, with treatment effects persisting over time.	Moderate	39

Chronic Urticaria

Author, year	Sub-condition	Conclusion	Certainty of Evidence	Total Number of Studies Included for Acupuncture
Yao et al,[91] 2016	None	Acupuncture might be effective and safe for chronic urticaria in relieving symptoms.	Very low to low	6

© 2022 Allen J et al. *JAMA Network Open.*

Constipation

Author, year	Sub-condition	Conclusion	Certainty of Evidence	Total Number of Studies Included for Acupuncture
Wang et al,[83] 2020	None	Acupuncture increased stool frequency, improved stool formation, and alleviated constipation symptoms.	Very low	8
		Acupuncture produced a significant benefit compared with polyethylene glycol and mosapride according to the Patient Assessment of Constipation Quality of Life (PAC-QOL) questionnaire.	Moderate	

Depression

Author, year	Sub-condition	Conclusion	Certainty of Evidence	Total Number of Studies Included for Acupuncture
Liu et al,[55] 2021	Post-stroke Depression	Acupuncture combined with conventional treatment could significantly reduce post-stroke depression. Acupuncture was safer than anti-depressants.	Very Low to Low	17
Smith et al,[57] 2018	None	The reduction in severity of depression was less when acupuncture was compared with control acupuncture than when acupuncture was compared with no treatment control.<br>The reduction in severity of depression with acupuncture given alone or in conjunction with medication versus medication alone is uncertain.<br>The effect of acupuncture compared with psychological therapy is unclear. Acupuncture did however have a positive effect on physical quality of life at the end of treatment when compared with sham acupuncture.	Very Low to Low	64
Smith et al,[56] 2019	Depression in Pregnancy	Acupuncture compared to control may reduce antenatal depression.	Moderate	2
Sorbero et al,[58] 2016	Major Depressive Disorder	Acupuncture may be superior to waitlist; limited evidence suggests a higher rate of responders with adjunctive acupuncture plus anti-depressants compared with anti-depressants alone.	Low	18

© 2022 Allen J et al. *JAMA Network Open.*

Dry Eye Syndrome

Author, year	Sub-condition	Conclusion	Certainty of Evidence	Total Number of Studies Included for Acupuncture
Jiang et al,[90] 2017	None	Acupuncture treatment could not improve the subjective symptoms for dry eye syndrome.	Very Low	2

Dysmenorrhea

Author, year	Sub-condition	Conclusion	Certainty of Evidence	Total Number of Studies Included for Acupuncture
Smith et al,[71] 2016	None	There is insufficient evidence to demonstrate whether acupuncture is effective in treating primary dysmenorrhea, and for most comparisons no data were available on adverse events.	Very Low to Low	32

Erectile Dysfunction

Author, year	Sub-condition	Conclusion	Certainty of Evidence	Total Number of Studies Included for Acupuncture
Lai et al,[87] 2019	None	When combination of acupuncture compared to tadalafil, the quality of evidence for cure rate and IIEF-5 scores was low. In comparison of other interventions and outcome assessments, the quality of evidence was mainly low or very low.	Very Low to Low	20

Fertility

Author, year	Sub condition	Conclusion	Certainty of Evidence	Total Number of Studies Included for Acupuncture
Coyle et al,[69] 2021	Assistive Reproductive Therapy	When compared with sham acupuncture, acupuncture performed at the time of embryo transfer does not result in better outcomes for live birth rate or for miscarriage rate.	High	6
Jo et al,[82] 2017	Polycystic Ovary Syndrome / Ovarian Hyperstimulation	Acupuncture may increase the clinical pregnancy rate and ongoing pregnancy rate and decrease the risk of Ovarian Hyperstimulation Syndrome in women with Polycystic Ovarian Syndrome undergoing in vitro fertilization or intracytoplasmic sperm injection.	Low	4

© 2022 Allen J et al. *JAMA Network Open.*

Author, year	Sub condition	Conclusion	Certainty of Evidence	Total Number of Studies Included for Acupuncture
Liu et al,[93] 2021	Pain management in Oocyte Retrieval	Compared with sham, there is moderate certainty of evidence that acupuncture complex analgesic therapy is more effective for pain management in oocyte retrieval. Compared to active treatment, there is low certainty of evidence that acupuncture complex analgesic therapy is more effective for pain management in oocyte retrieval.	Moderate<br>Low	14
Lim et al,[68] 2019	Anovulatory Infertility	There was no evidence of any clinically relevant differences in live birth rate, multiple pregnancy rate, ovulation rate, clinical pregnancy rate, and miscarriage rate in sham vs acupuncture.<br>We were uncertain whether acupuncture improved ovulation rate compared to active treatment.	Low<br>Very Low	8

Fibromyalgia

Author, year	Sub-condition	Conclusion	Certainty of Evidence	Total Number of Studies Included for Acupuncture
Kim et al,[34] 2019	Pain, Fatigue, Sleep Quality	Verum acupuncture is more effective than sham acupuncture for pain relief, improving sleep quality, and improving general status in fibromyalgia syndrome posttreatment.	Moderate to High	10
Zhang et al,[35] 2019	None	Compared with sham, real acupuncture was more effective in reducing pain and improving quality of life after treatment in the short term.<br>At follow-up in the long term, the effect of acupuncture was also superior to that of sham acupuncture.	Low to Moderate<br>Low	12

Functional Dyspepsia

Author, year	Sub-condition	Conclusion	Certainty of Evidence	Total Number of Studies Included for Acupuncture
Pang et al,[72] 2016	None	Acupuncture therapy has a similar effect for functional dyspepsia in comparison with sham acupuncture.<br>Acupuncture therapy is superior to medication (prokinetic agents) in improving the symptoms and quality of life of functional dyspepsia patients.	Low<br>Low	16

© 2022 Allen J et al. *JAMA Network Open.*

Gastroparesis

Author, year	Sub-condition	Conclusion	Certainty of Evidence	Total Number of Studies Included for Acupuncture
Kim et al,[88] 2017	None	There is short-term benefit with acupuncture alone or acupuncture combined with gastrokinetic drugs compared with the drug alone, in terms of the proportion of people who experienced improvement in diabetic gastroparesis.	Very low	29

Headache

Author, year	Sub-condition	Conclusion	Certainty of Evidence	Total Number of Studies Included for Acupuncture
Giovanardi et al,[36] 2020	Migraine	Acupuncture is mildly more effective and much safer than medication for the prophylaxis of migraine.	Moderate	9
Linde et al,[40] 2016	Tension-type Headache	Acupuncture reduces headache frequency over usual care and sham.	Moderate	12
Linde et al,[39] 2016	Migraine	Compared with no acupuncture, acupuncture was associated with a moderate reduction of headache frequency over no acupuncture after treatment.<br>Comparison with sham, both after treatment and at follow-up, acupuncture was associated with a small but statistically significant frequency reduction over sham.<br>Compared with prophylactic drug treatment, acupuncture reduced migraine frequency significantly more than drug prophylaxis after treatment.	Moderate<br>Moderate<br>Moderate	22
Xu et al,[38] 2018	Migraine Headache without Aura	Acupuncture had a significant advantage over medication in reducing frequency of migraine, pain score, and effective rate. Acupuncture also had a significant advantage over sham acupuncture in reducing frequency of migraine and pain score.	Very Low to Low	14
Yun et al,[37] 2020	Occipital Neuralgia	Acupuncture was more effective that medication at reducing VAS pain.<br>Acupuncture was more effective than medication on the total effective rate.	Very Low<br>Low	11

© 2022 Allen J et al. *JAMA Network Open.*

Herpes Zoster

Author, year	Sub-condition	Conclusion	Certainty of Evidence	Total Number of Studies Included for Acupuncture
Cui et al,[74] 2021	None	When compared with antiviral therapy, acupuncture was associated with a significant reduction in pain, a significant reduction in incrustation time, and a significant reduction in decrustation time.	Low	21
		Compared with active treatment, acupuncture was associated with reduction on the overall incidence of post-herpetic neuralgia	Moderate	

Hyperemesis Gravidum

Author, year	Sub-condition	Conclusion	Certainty of Evidence	Total Number of Studies Included for Acupuncture
Sridharan et al,[19] 2020	None	Acupuncture was associated with better control of symptoms than standard care.	Very low	1

Inflammatory Bowel Disease

Author, year	Sub-condition	Conclusion	Certainty of Evidence	Total Number of Studies Included for Acupuncture
Wang et al,[76] 2020	None	Acupuncture may be more effective in treating ulcerative colitis compared to conventional medicine (metronidazole combined with sulfasalazine).	Low to Moderate	13

© 2022 Allen J et al. *JAMA Network Open.*

Insomnia

Author, year	Sub-condition	Conclusion	Certainty of Evidence	Total Number of Studies Included for Acupuncture
Cao et al,[60] 2019	Primary Insomnia	Acupuncture might result in improvement compared to no treatment on Pittsburgh Sleep Quality Index scores and appears safe.	Very Low to Low	73
Kwon et al,[59] 2020	Insomnia in Elderly	Using Pittsburgh Sleep Quality Index score, acupuncture and acupuncture combined with relaxation were both more effective in improving sleep quality compared to relaxation alone.	Very Low to Moderate	13
Choi et al,[66] 2017	Cancer-related Insomnia	Acupuncture may be superior with sham acupuncture, drugs or hormones therapy.	Low	3

Irritable Bowel Syndrome

Author, year	Sub-condition	Conclusion	Certainty of Evidence	Total Number of Studies Included for Acupuncture
Guo et al,[77] 2020	None	Compared with loperamide, acupuncture showed more effectiveness in weekly defecation. Compared to dicetel, acupuncture produced more significant effect related to the total symptom score and IBS Symptom Severity Scale.	Low to Moderate	31

Lateral Elbow Pain

Author, year	Sub-condition	Conclusion	Certainty of Evidence	Total Number of Studies Included for Acupuncture
Navarro-Santana et al,[20] 2020	None	Evidence suggests positive effects of acupuncture, but not electro-acupuncture, for pain, related disability, and strength, in lateral epicondylalgia of musculoskeletal origin, in the short term.	Very Low to Low	14

© 2022 Allen J et al. *JAMA Network Open.*

Menopause

Author, year	Sub-condition	Conclusion	Certainty of Evidence	Total Number of Studies Included for Acupuncture
Dodin et al,[78] 2013	None	When acupuncture was compared with sham acupuncture, there was no evidence of any difference in their effect on hot flushes. When acupuncture was compared with no treatment, there appeared to be a benefit from acupuncture, but acupuncture appeared to be less effective than HT.	Very Low to Low	16

Mixed Not Specified Pain

Author, year	Sub-condition	Conclusion	Certainty of Evidence	Total Number of Studies Included for Acupuncture
Chia et al,[18] 2018	Painful Conditions in Emergency Department	Acupuncture was superior with sham acupuncture, more effective than intravenous morphine, comparable to conventional Emergency Department treatment, and superior to standard Emergency Department care alone when used on an adjuvant basis.	Low	6
Liu et al,[16] 2019	Post-stroke Shoulder-Hand Syndrome	Acupuncture therapy seems effective for motor function, pain relief, and activities of daily living in stroke patients with mild Shoulder-hand Syndrome, when it is used in combination with rehabilitation.	Low	38
Xiang et al,[51] 2017	Immediate Pain Relief in Musculoskeletal Pain Conditions	Acupuncture was associated with a greater immediate pain relief effect compared with sham acupuncture.	Moderate	13
		Acupuncture was associated with greater immediate pain relief effect when compared to analgesic injections.	Low	

© 2022 Allen J et al. *JAMA Network Open.*

Neck Pain

Author, year	Sub-condition	Conclusion	Certainty of Evidence	Total Number of Studies Included for Acupuncture
Skelly et al,[29] 2020	Chronic Neck Pain	Acupuncture was associated with small improvements in short-term and intermediate-term function versus sham acupuncture, a placebo (sham laser), or usual care.	Low	11
		There were no differences in pain in trials comparing acupuncture with sham acupuncture or placebo interventions in the short term.	Low	
		There was insufficient evidence to draw conclusions regarding short-term function or pain for acupuncture versus NSAIDs.	Low	
		No serious adverse events were reported in 6 trials reporting harms.	Low	

Obstructive Sleep Apnea

Author, year	Sub-condition	Conclusion	Certainty of Evidence	Total Number of Studies Included for Acupuncture
Wang et al,[85] 2020	None	Acupuncture therapy is effective for obstructive sleep apnea patients in reducing apnea-hypopnea index (low certainty) and Epworth Sleepiness Score (very low certainty) and in improving the lowest oxygen saturation (very low) of various severities, especially in moderate and severe obstructive sleep apnea patients.	Very Low to Low	9

© 2022 Allen J et al. *JAMA Network Open.*

Osteoarthritis

Author, year	Sub-condition	Conclusion	Certainty of Evidence	Total Number of Studies Included for Acupuncture
Manheimer et al,[52] 2018	Hip pain	Acupuncture probably has little or no effect in reducing pain or improving function relative to sham acupuncture in people with hip osteoarthritis.	Moderate	6
Skelly et al,[29] 2020	Knee pain	There were no differences between acupuncture versus control interventions (sham acupuncture, waitlist, or usual care) on function in the intermediate term	Low	9
		There were no clinically meaningful differences between acupuncture versus control interventions (sham acupuncture, waitlist, or usual care) on pain in the intermediate term.	Moderate	

Other Acute Pain

Author, year	Sub-condition	Conclusion	Certainty of Evidence	Total Number of Studies Included for Acupuncture
Chou et al,[28] 2020	Post-operative Pain	There is inconsistent evidence on acupuncture's effect on pain intensity when compared with sham. Acupuncture may be associated with decrease analgesic use after 1 day compared to usual care.	Very Low	2
Chou et al,[28] 2020	Dental Surgical Pain	There is insufficient evidence of acupuncture's effect on post-operative pain compared with sham acupuncture.	Very Low	1
Chou et al,[28] 2020	Kidney Stone	Acupuncture was not effective in reducing pain intensity vs medication for kidney stone.	Low	1

Other Chronic Pain - Various

Author, year	Sub-condition	Conclusion	Certainty of Evidence	Total Number of Studies Included for Acupuncture
Eccleston et al,[8] 2017	Chronic Non-cancer Pain	There is no evidence for the efficacy or safety of electro-acupuncture for reducing prescribed opioid use in chronic pain.	Very Low	1

© 2022 Allen J et al. *JAMA Network Open.*

Other Specific

Author, year	Sub-condition	Conclusion	Certainty of Evidence	Total Number of Studies Included for Acupuncture
Zhou et al,[75] 2020	Improvement of Cognitive Impairment After Stroke	Acupuncture was effective in improving PSCI (post-stroke cognitive impairment) compared to no treatment or sham.	Moderate	3

Pelvic Pain

Author, year	Sub-condition	Conclusion	Certainty of Evidence	Total Number of Studies Included for Acupuncture
Franco et al,[47] 2019	Chronic Prostatitis/ Chronic Pelvic Pain Syndrome	Acupuncture probably reduced prostatitis symptoms (compared with sham).<br>Acupuncture may have reduced prostatitis symptoms compared with medical treatment	Moderate<br>Moderate	6

Peripheral Neuropathy

Author, year	Sub-condition	Conclusion	Certainty of Evidence	Total Number of Studies Included for Acupuncture
Ju et al,[41] 2017	None	There is insufficient evidence to support or refute the use of acupuncture for neuropathic pain in general or for any specific neuropathic pain condition when compared with sham acupuncture or other active therapies.	Very Low to Low	6

Peripheral Neuropathy (Diabetic)

Author, year	Sub-condition	Conclusion	Certainty of Evidence	Total Number of Studies Included for Acupuncture
Amato et al, [9] 2019	None	Evidence for acupuncture was insufficient for diabetic peripheral neuropathy.	Very Low	1

© 2022 Allen J et al. *JAMA Network Open.*

Post-Herpetic Neuralgia

Author, year	Sub-condition	Conclusion	Certainty of Evidence	Total Number of Studies Included for Acupuncture
Pei et al,[42] 2019	None	Acupuncture was more effective in reducing post-herpetic neuralgia pain intensity compared to control.	Low to Moderate	4

Post-Operative Pain

Author, year	Sub-condition	Conclusion	Certainty of Evidence	Total Number of Studies Included for Acupuncture
Tedesco et al,[45] 2017	None	Acupuncture reduced or delayed opioid consumption compared with sham or no treatment.	Low to Moderate	4
Yin et al,[44] 2020	None	Compared to active treatment, acupuncture may improve the overall symptoms of Postcholecystectomy syndrome (PCS).	Low to Moderate	14
Zimpel et al,[43] 2020	Post-caesarean Pain	We are very uncertain if acupuncture (versus no treatment) or acupuncture plus analgesia (versus placebo plus analgesia) has any effect on pain because the quality of evidence is very low. Acupuncture plus analgesia (versus analgesia) may reduce pain at 12 hours and 24 hours.	Very Low	4

Post-Operative Cognitive Dysfunction

Author, year	Sub-condition	Conclusion	Certainty of Evidence	Total Number of Studies Included for Acupuncture
Tang et al,[27] 2021	None	Acupuncture may successfully treat and/or prevent post-operative cognitive dysfunction.	Very Low	16

© 2022 Allen J et al. *JAMA Network Open.*

Post-Stroke Dysarthria

Author, year	Sub-condition	Conclusion	Certainty of Evidence	Total Number of Studies Included for Acupuncture
Xie et al,[84] 2020	None	The combination of acupuncture and speech rehabilitation training may improve total response rate of stroke patients with dysarthria.	Low	17

Post-Stroke Urinary Incontinence

Author, year	Sub-condition	Conclusion	Certainty of Evidence	Total Number of Studies Included for Acupuncture
Thomas et al,[23] 2019	None	Acupuncture shows beneficial effect as adjunctive treatment for people mainly with psychogenic ED.	Very Low to Low	5

Post-Traumatic Stress Disorder

Author, year	Sub-condition	Conclusion	Certainty of Evidence	Total Number of Studies Included for Acupuncture
Grant et al,[61] 2018	None	Needle acupuncture reduces PTSD and depressive symptoms at follow-up compared to passive controls, treatment-as-usual, and active interventions. No significant differences were observed between acupuncture and comparators for other outcomes.	Very Low to Low	7

Pregnancy

Author, year	Sub-condition	Conclusion	Certainty of Evidence	Total Number of Studies Included for Acupuncture
Liddle et al,[81] 2015	Low Back and Pelvic Pain	There was evidence from single studies that acupuncture significantly improves evening pelvic pain better than stabilizing exercise or usual prenatal care.	Moderate	4
		There is evidence suggesting that acupuncture is better than physiotherapy at relieving evening low back and pelvic pain and related functional disability, and improves pain, but not women's ability to carry out daily activities, when started at 26- rather than 20-weeks' gestation.	Low	

© 2022 Allen J et al. *JAMA Network Open.*

Premenstrual Syndrome

Author, year	Sub-condition	Conclusion	Certainty of Evidence	Total Number of Studies Included for Acupuncture
Armour et al,[79] 2018	None	Acupuncture may reduce overall mood and physical PMS symptoms when compared with sham. There was not enough evidence to determine the safety of acupuncture.	Low	4

Primary Ovarian Insufficiency

Author, year	Sub-condition	Conclusion	Certainty of Evidence	Total Number of Studies Included for Acupuncture
Jo et al,[82] 2015	Resumption of Menses	Acupuncture was better than comparison treatments in the resumption of menses.	Low	6
		There are insufficient data to reach conclusions about the effect of acupuncture on symptoms.	Very Low	

Primary Trigeminal Neuralgia

Author, year	Sub-condition	Conclusion	Certainty of Evidence	Total Number of Studies Included for Acupuncture
Hu et al,[46] 2019	None	Acupuncture might have some positive effects for primary trigeminal neuralgia.	Very Low to Low	33

Schizophrenia

Author, year	Sub-condition	Conclusion	Certainty of Evidence	Total Number of Studies Included for Acupuncture
Shen et al,[62] 2014	None	Limited evidence suggests that acupuncture may have some antipsychotic effects as measured on global and mental state with few adverse effects.	Very Low to Low	30

© 2022 Allen J et al. *JAMA Network Open.*

Shoulder Pain

Author, year	Sub-condition	Conclusion	Certainty of Evidence	Total Number of Studies Included for Acupuncture
Yuan et al,[30] 2016	None	Acupuncture is superior to sham acupuncture in relief of pain.	High	5
Ben-Arie et al,[53] 2020	Frozen Shoulder	Acupuncture could be safe and effective for pain reduction, restoring shoulder function, and restoring flexion ROM for frozen shoulder patients in the short term and midterm.	Very Low	13

Stress Urinary Incontinence

Author, year	Sub-condition	Conclusion	Certainty of Evidence	Total Number of Studies Included for Acupuncture
Zhong et al,[14] 2020	None	There is moderate level of evidence for the effectiveness of electroacupuncture for women with stress urinary incontinence compared with sham, but very low to low level of evidence for manual acupuncture and other outcomes.	Very low to Moderate	10

Stroke

Author, year	Sub-condition	Conclusion	Certainty of Evidence	Total Number of Studies Included for Acupuncture
Liu et al, [26] 2015	None	Moderate-level certainty of evidence for effectiveness of electroacupuncture plus western conventional treatment (WCTs) for improving total clinical effectiveness rate compared with WCTs, and for effectiveness of electroacupuncture for improving the Chinese Stroke Recovery Scale.	Moderate	18
		High-level certainty of evidence showing better outcomes associated with electroacupuncture plus western conventional treatments for improving National Institutes of Health Stroke Scale, Barthel Index and the Fugl–Meyer Assessment total.	High	
Xu et al, [89] 2018	None	When compared with any control, findings of lower odds of death or dependency at the end of follow-up and over the long term, or better global neurologic deficit score, or difference in death or institutional care, in the acupuncture group were uncertain.	Very low to low	33

© 2022 Allen J et al. *JAMA Network Open.*

Stewart et al,[21] 2018	Post-stroke activities of daily living	No evidence was found to support use of acupuncture to benefit older stroke survivors' disability.	Low	11

Substance Use Disorder

Author, year	Sub-condition	Conclusion	Certainty of Evidence	Total Number of Studies Included for Acupuncture
Chen et al,[63] 2018	Opioid Use Disorder	Acupuncture may be effective for alleviating some symptoms compared with sham and no treatment.<br><br>There was insufficient evidence to suggest better effect of acupuncture compared medication.	Very Low to low<br><br>Moderate	9
White et al,[64] 2014	Tobacco Use Disorder	Compared with sham, acupuncture resulted in greater short-term smoking cessation.	Moderate	19
Ge et al,[65] 2020	Illicit drug withdrawal syndrome	Acupuncture may alleviate illicit drug withdrawal syndrome.	Very low to low	30

Temporomandibular Joint Dysfunction

Author, year	Sub-condition	Conclusion	Certainty of Evidence	Total Number of Studies Included for Acupuncture
Yuan et al,[30] 2016	None	Real acupuncture showed a favorable effect on pain relief compared with sham.	Moderate	13

Tinnitus

Author, year	Sub-condition	Conclusion	Certainty of Evidence	Total Number of Studies Included for Acupuncture
Savage et al,[22] 2014	None	Unclear if acupuncture is effective in people with tinnitus.	Low	1

© 2022 Allen J et al. *JAMA Network Open.*

Urinary Tract Infection

Author, year	Sub-condition	Conclusion	Certainty of Evidence	Total Number of Studies Included for Acupuncture
Qin et al,[17] 2020	None	Acupuncture may be more effective than no treatment in preventing recurrence.	Low	3
		Acupuncture more effective than sham in preventing recurrence.	Moderate	

© 2022 Allen J et al. *JAMA Network Open.*

eAppendix 4. Evidence Map for Adverse Events

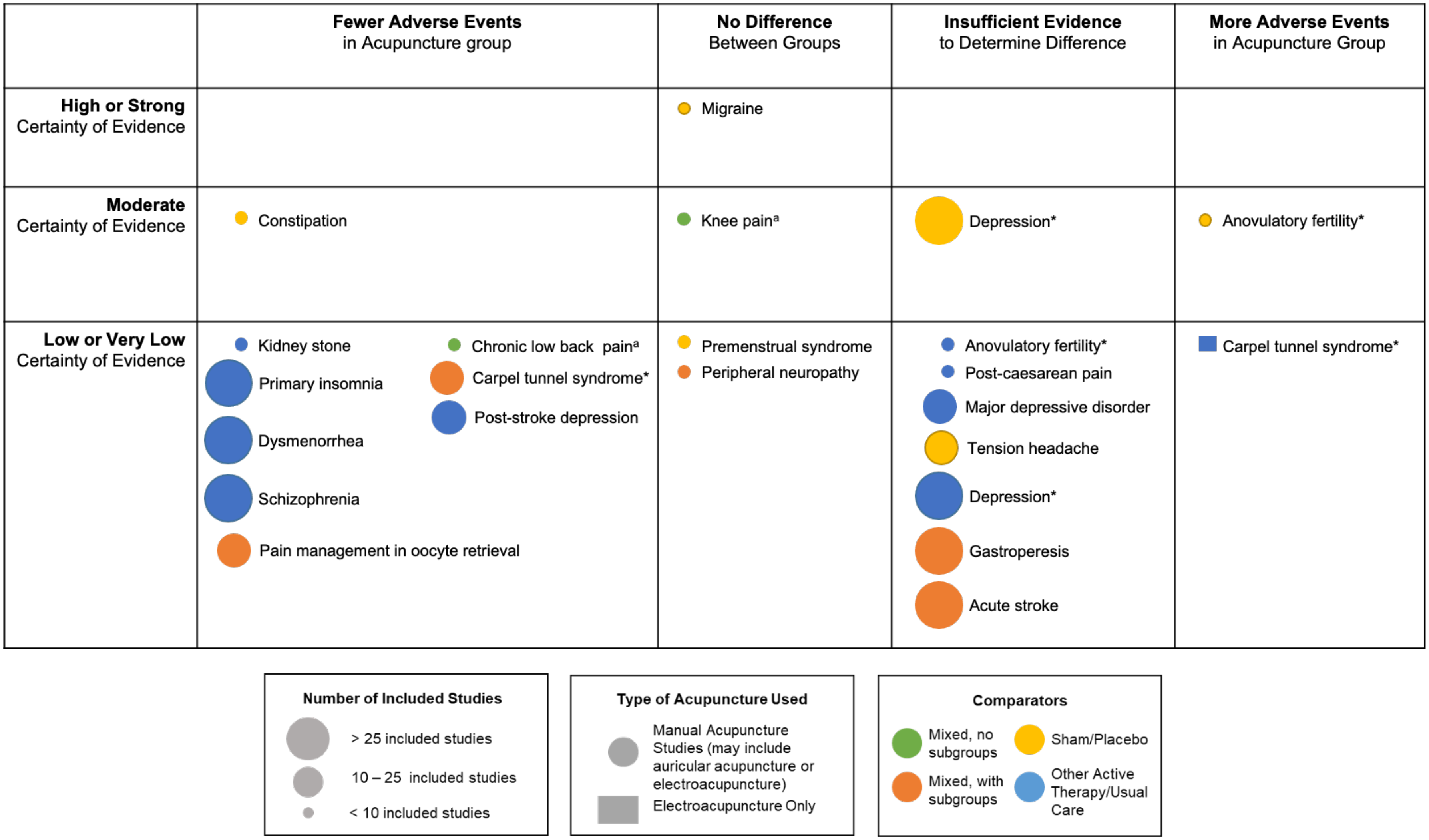

[a] Review included distinct conclusions about separate conditions, therefore it appears more than once.
[*] Review included distinct conclusions about separate comparators, therefore it appears more than once.

© 2022 Allen J et al. *JAMA Network Open.*

eAppendix 5. Certainty of Evidence Conclusions for Adverse Events in Reviews Included in Map

High Certainty of Evidence for Adverse Events

Author, year	Condition	Sub condition	Certainty of Evidence Conclusion
Linde et al,[39] 2016	Headache	Migraine	There is no difference in the number of participants experiencing serious adverse events between acupuncture and sham acupuncture.

Moderate Certainty of Evidence for Adverse Events

Author, year	Condition	Sub condition	Certainty of Evidence Conclusion
Smith et al,[57] 2018	Depression	None	It is unclear whether there are differences in the risk of adverse events between persons receiving acupuncture or sham acupuncture.
Lim et al,[68] 2019	Fertility	Anovulatory infertility	True acupuncture probably worsens adverse events compared with sham acupuncture.
Skelly et al,[29] 2020	Osteoarthritis	Knee pain	There was no difference in the risk of serious adverse events between any form of acupuncture and the control group.
Wang et al,[83] 2021	Constipation	None	Acupuncture is safer than medication.

Low or Very Low Certainty of Evidence for Adverse Events

Author, year	Condition	Sub condition	Certainty of Evidence Conclusion
Skelly et al,[29] 2020	Back pain	Chronic low back pain	Serious adverse events were rare with acupuncture and control.
Choi et al,[25] 2018	Carpal tunnel syndrome	None	Acupuncture was associated with fewer or no serious adverse events compared to active or sham groups.
Choi et al,[25] 2018	Carpal tunnel syndrome	None	Electro-acupuncture was associated with more adverse events when compared with night splints.
Liu et al,[55] 2021	Depression	Post-stroke depression	Acupuncture was associated with fewer adverse events than antidepressants, but there was no significant difference in the occurrence of adverse events between the combination of acupuncture and conventional treatments versus conventional treatments.
Smith et al,[57] 2018	Depression	None	The risk of adverse events with acupuncture is unclear, as most trials did not report adverse events adequately.
Sorbero et al,[58] 2016	Depression	Major Depressive Disorder	Insufficient data to determine if there are differences between groups for adverse events.
Smith et al,[71] 2016	Dysmenorrhea	None	Adverse events were less common in the acupuncture group compared to NSAID.
Lim et al,[68] 2019	Fertility	Anovulatory infertility	Insufficient data to determine if there are differences between acupuncture and usual care or active treatment for adverse events.

© 2022 Allen J et al. *JAMA Network Open.*

Author, year	Condition	Sub condition	Certainty of Evidence Conclusion
Liu et al,[93] 2021	Fertility	Pain management in oocyte retrieval	Acupuncture combined with analgesia decreased adverse effects compared to using analgesia alone.
Kim et al,[88] 2017	Gastroparesis	None	Any difference in adverse events between acupuncture and sham or active therapy is very uncertain.
Linde et al,[40] 2016	Headache	Tension-type Headache	There is no evidence to conclude that adverse events differ between patients receiving acupuncture or sham acupuncture.
Cao et al,[60] 2019	Insomnia	Primary Insomnia	Fewer adverse events from acupuncture than Western medications.
Chou et al,[28] 2020	Other acute pain	Kidney Stone	For kidney stone pain, acupuncture vs NSAID or acetaminophen, there were few adverse events in 1 trial.
Ju et al,[41] 2017	Peripheral neuropathy	None	No clear differences were observed between acupuncture and sham or active groups.
Zimpel et al,[43] 2020	Post-operative pain	Post-Caesarean pain	It is uncertain whether acupuncture (vs no treatment) or acupuncture plus analgesia (vs analgesia) has any effect on the risk of adverse effects.
Armour et al,[79] 2018	Premenstrual syndrome	None	There was insufficient evidence to determine whether there was a difference between acupuncture and sham or no treatment in the rates of adverse events.
Shen et al,[62] 2014	Schizophrenia	None	Acupuncture compared with standard antipsychotic treatment alone; adverse effects were less for the acupuncture group.
Xu et al,[89] 2018	Stroke	None	When acupuncture was compared with sham acupuncture, findings for adverse events were uncertain.

© 2022 Allen J et al. *JAMA Network Open.*
