# Supplementary material for: Use of Acupuncture for Adult Health Conditions, 2013 to 2021: A Systematic Review
Source: JAMA Netw Open. 2022 Nov 23;5(11):e2243665. doi: 10.1001/jamanetworkopen.2022.43665 (PMC9685495; doi:10.1001/jamanetworkopen.2022.43665)
Supplement: Supplement 2. — Data Sharing Statement [file jamanetwopen-e2243665-s002.pdf]

Data Sharing Statement

Allen. Use of Acupuncture for Adult Health Conditions, 2013 to 2021. *JAMA Netw Open*.
Published November 23, 2022. doi:10.1001/jamanetworkopen.2022.43665

Data
Data available: No
